# Seabird trajectories map onto a reduced optimal-control bound for dynamic soaring

**Published:** 2026-04-15

**Authors:** Louis González, Saad Bhamla

**Affiliations:** a School of Chemical & Biomolecular Engineering, Georgia Institute of Technology, Atlanta, USA; b BioFrontiers Institute, University of Colorado, Boulder, USA; c Department of Chemical & Biological Engineering, University of Colorado, Boulder, USA

**Keywords:** Dynamic soaring, Optimal control, Physics of life, Bird migration, Pareto frontier

## Abstract

Dynamic soaring allows seabirds to harvest mechanical energy from vertical wind shear, but field trajectories lack a benchmark for comparing flight performances across species. We derive a reduced lower bound on transport effort from a simplified Hamilton-Jacobi-Bellman optimal-control model in which slow flight incurs an induced-drag penalty, fast flight incurs a dissipative penalty, and wind shear supplies an effective energetic subsidy. After species-specific normalization of transport speed and an accelerometer-based effort proxy, we map wandering albatrosses, Cory’s shearwaters, and Eurasian oystercatchers into a common reduced speed–effort plane and estimate their empirical lower frontiers. The albatross frontier lies closest to the reduced bound, consistent with near-optimal wind-energy harvesting. The shearwater frontier is systematically displaced above it, and oystercatchers occupy a distinct non-soaring regime. The resulting framework places specialist dynamic soaring, mixed flap-gliding, and non-soaring flight in a common mechanical representation and provides a reduced benchmark for comparing wind-assisted flight across species using field trajectories.

Dynamic soaring allows birds to extract mechanical energy from vertical wind shear and sustain long-range flight with little active propulsion (1, 2). This flight mode is especially prominent in Procellariiform seabirds (2–5), where wandering albatrosses can cover more than 10,000 km in a single foraging trip (6). Recent work has shown that birds exploit wind shear in structured and often optimized ways (7, 8), but field data still lack a benchmark that places observed trajectories relative to a minimum-cost boundary.

To test such a boundary, we analyze public GPS tracking and accelerometry records from three bird groups spanning a gradient in dynamic-soaring capability: 44 wandering albatrosses (*Diomedea exulans*) that exploit boundary-layer shear over the Southern Ocean (9), a comparison cohort of Cory’s shearwater (*Calonectris borealis*) drawn from a 24-bird Movebank accession and representing mixed flap-gliding with wind-shear use (7, 10), and a 20-bird subset of Eurasian oystercatcher (*Haematopus ostralegus*), continuously flapping shorebirds used here as a non-soaring negative control (11). From each dataset, we derive ground-referenced transport variables and accelerometry-based effort measures and, after species-specific normalization, map them into a common reduced speed-effort plane. For the reduced comparison developed here, these observables are sufficient to place specialist soaring, mixed flap-gliding, and non-soaring flight in a common frame.

## Albatross trajectories define an empirical transport–effort frontier consistent with wind-assisted flight.

**Fig. 1.**
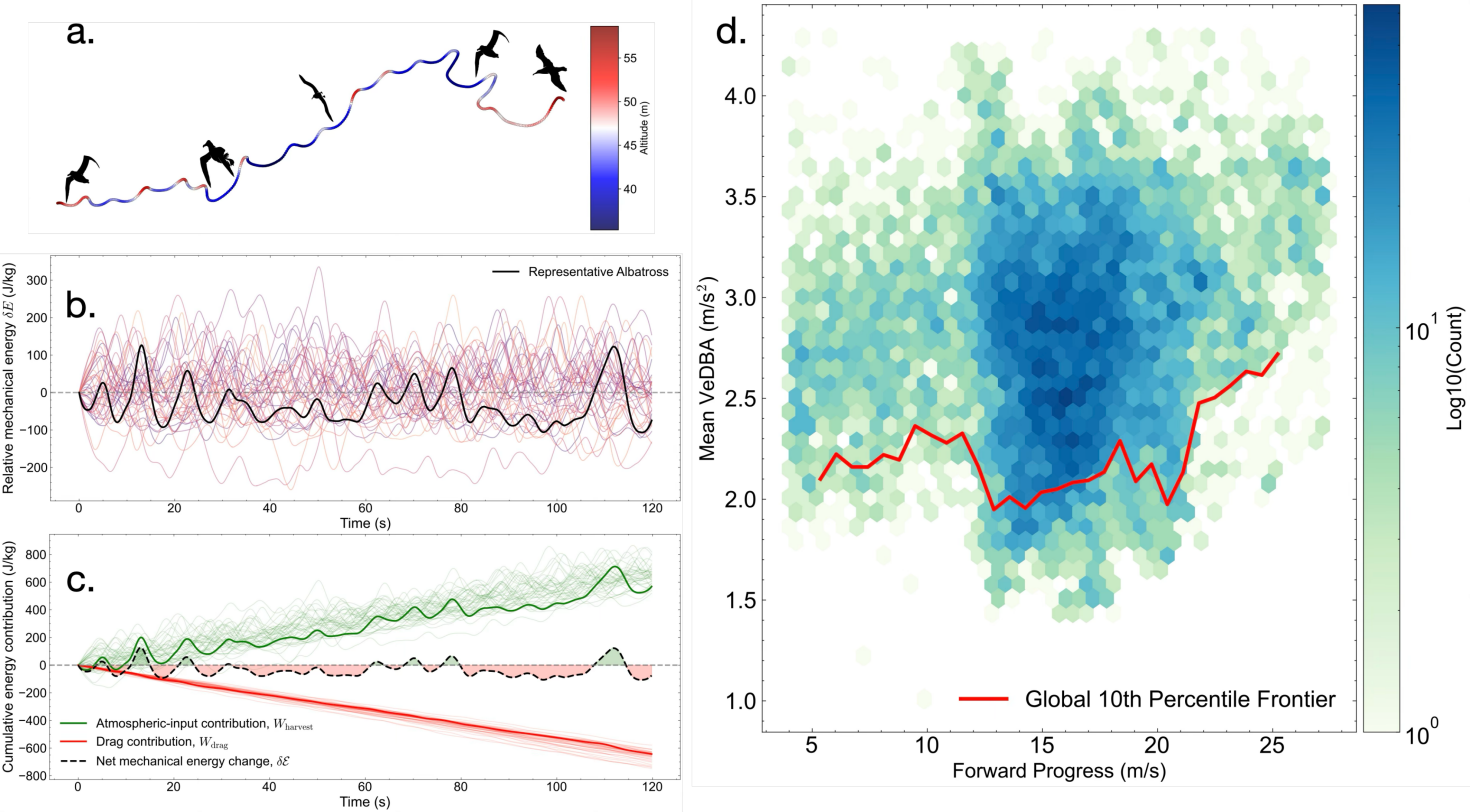
Albatross trajectories show cyclic energy exchange and define an empirical transport–effort frontier. **a.** Representative wandering albatross, colored by altitude, showing repeated turning and climb–descent cycles typical of dynamic soaring. **b.** Relative ground-frame specific mechanical energy $\delta E(t)=E(t)-E\left(t_{0}\right)$ for multiple 120-s trajectory windows, with one representative window highlighted. **c.** Signed cumulative atmospheric-input term, $W_{\text{drag}}(t)$, and signed cumulative atmospheric-input term, $W_{\text{harvest}}(t)$, whose sum gives the net energy change under the gliding approximation. **d.** Global hexbin density in the plane of net transport speed and mean VeDBA, with the red curve denoting the global 10th-percentile lower frontier.

Fig. 1a displays a representative albatross trajectory, colored by altitude, with the repeated turning and climbing-descent cycles characteristic of dynamic soaring. Continuous tracks were partitioned into 120-s overlapping windows, and for each window, we computed the ground-frame specific mechanical energy $E(t)=\frac{1}{2}u{\left(t\right)}^{2}+gz(t)$, where u(t) is ground speed and z(t) is altitude. Figure 1b plots the relative energy $\delta E(t)=E(t)-E\left(t_{0}\right)$ across many windows, with one representative trace highlighted. The oscillatory pattern reflects repeated exchanges between kinetic and potential energy during sheared flight.

To interpret these fluctuations, we estimate a cumulative drag work term using a quasi-steady gliding model and infer the corresponding cumulative atmospheric-input term (or wind-mediated gain) $W_{\text{harvest}}(t)=\delta E(t)-W_{\text{drag}}(t)$ under the approximation that sustained muscular input is small over these gliding-dominated windows. Figure 1c shows these two contributions, confirming them to be of comparable magnitude and opposite sign, consistent with a near-steady energy budget maintained by shear exploitation (1, 4). Because the ambient wind field is not reconstructed pointwise, $W_{\text{harvest}}$ is interpreted as an effective atmospheric-input observable rather than a direct measurement of aerodynamic wind work. Its magnitude is nonetheless comparable to the inferred drag loss, consistent with atmospheric energy input offsetting dissipation during sustained transport.

**Fig. 2.**
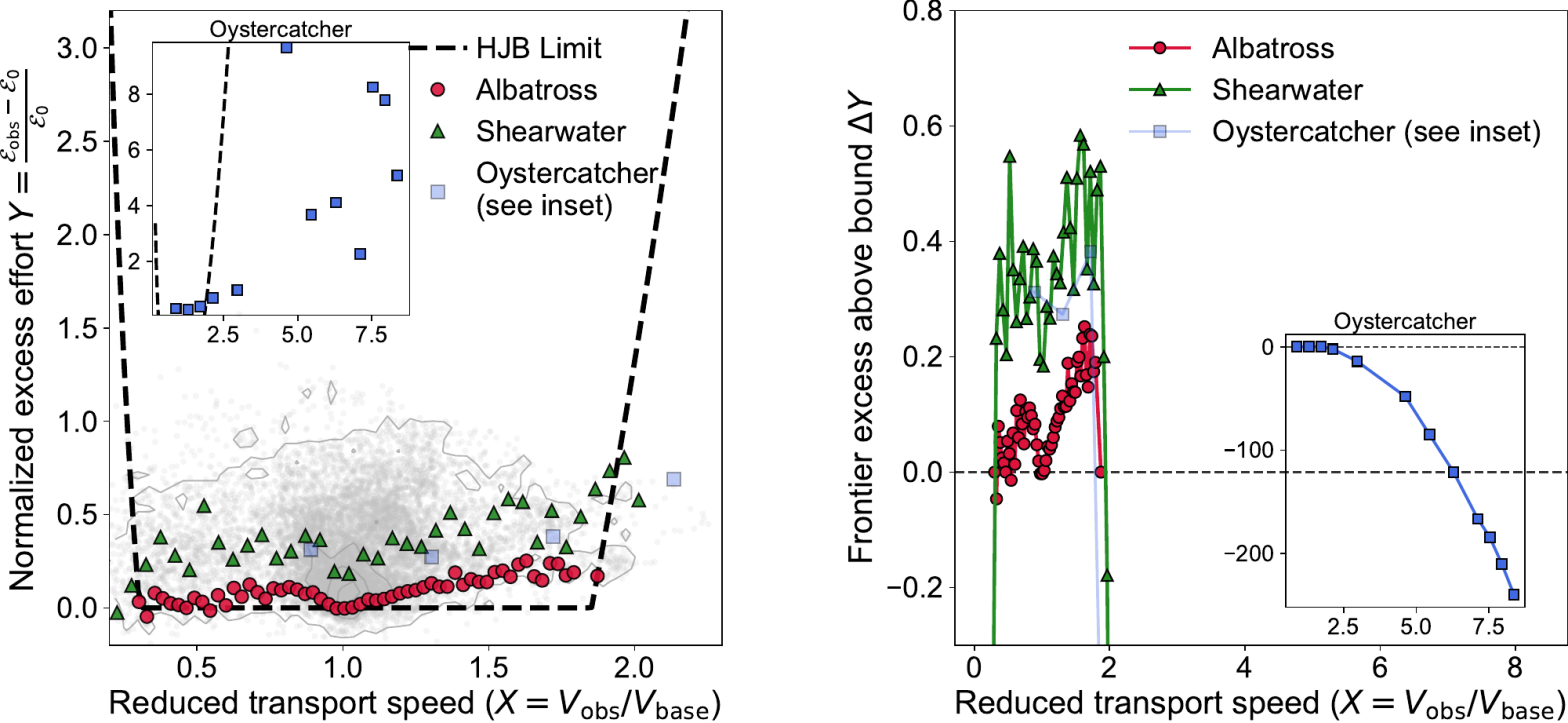
A reduced bound separates dynamic-soaring from non-soaring flight. **a.** Species-specific 10th-percentile lower frontiers in the reduced speed–effort plane, where X is reduced transport speed and $Y=\left(E_{\text{obs}}-E_{0}\right)/E_{0}$ is normalized excess effort based on VeDBA. Gray contours show the bird-by-bird normalized albatross population points derived from Fig. 1d. The dashed curve shows the reduced HJB bound, whose functional form is derived from the optimal-control model. Albatross frontiers cluster closest to the reduced benchmark, shearwaters are systematically higher, and oystercatchers lie far outside the soaring-compatible region (inset). Panel **b.** is the vertical residual or, in other words, the excess above the bound $\text{Δ}Y=Y_{\text{frontier}}-Y_{\text{HJB}}(X)$ as a function of X. The ordering of residuals quantifies species differences in proximity to the minimum-cost soaring regime.

Fig. 1d then collapses all retained albatross windows into the empirical plane of net transport speed versus mean vectorial dynamic body acceleration (VeDBA), an accelerometer-based proxy for locomotor effort (12). The hexbin cloud shows the population distribution, and the red curve marks the 10th-percentile lower binned frontier. This lower frontier serves as our key empirical reference: it summarizes the best-observed transport-effort trade-off in the albatross dataset. It provides the albatross baseline for reduced cross-species comparison in Fig. 2.

## A reduced HJB benchmark organizes cross-species transport–effort frontiers.

To compare species with different absolute flight speeds and effort scales, we transformed each dataset into the reduced variables $X=V_{\text{obs}}/V_{\text{base}}$, $Y=\left(E_{\text{obs}}-E_{0}\right)/E_{0}$, where $V_{\text{base}}$ is a low-effort reference speed and $E_{0}$ is a low-effort baseline effort proxy. For the albatross population, this normalization was applied bird by bird to the windowed transport-progress and mean-VeDBA quantities from Fig. 1d; for shearwaters and oystercatchers, it was applied to retained GPS–acceleration observations after species-specific preprocessing (see SI). The resulting species frontiers are shown in Fig. 2a.

The dashed curve is the reduced Hamilton-Jacobi-Bellman (HJB) (2, 8) form

$$Y_{\text{HJB}}(X)={\left[\frac{a}{X^{2}}+bX^{2}-WX\right]}_{+},$$

whose functional form follows from a simplified optimal-control model of along-track transport in prescribed shear. In this reduced description, the $a/X^{2}$ term captures the penalty for slow flight, the $bX^{2}$ term captures the highspeed dissipative cost, and the −WX term represents an effective wind-energy subsidy supplied by shear exploitation. Because Y is a normalized excess-effort quantity relative to a low-effort baseline, ${\left[\cdot \right]}_{+}$ enforces clipping below zero. The functional form is derived from the model, whereas the numerical parameters (a,b,W) are estimated from the albatross frontier.

Within the soaring-compatible range, the albatross frontier (red circles) lies closest to this reduced benchmark, whereas the shearwater frontier (green triangles) is systematically displaced above it. This ordering is consistent with albatrosses operating nearest the reduced minimum-cost regime predicted by optimal-control theory and with shearwaters occupying a mixed flap-gliding regime that uses wind shear but remains more effort-intensive. The theory derives the reduced shape; the empirical normalization maps the VeDBA-based effort onto that reduced form. Oystercatchers (blue squares) occupy a distinct, continuously flapping regime and are shown separately in the inset rather than as part of the soaring branch.

Figure 2b summarizes the comparison with the inferred vertical offset $\text{Δ}Y=Y_{\text{frontier}}-Y_{\text{HJB}}(X)$. For albatrosses and shearwaters, values near zero indicate proximity to the reduced optimum, while positive values indicate displacement above it. The negative values in the oystercatcher inset reflect the upturn of the fitted curve at large X. Across the retained range, albatrosses show the smallest residuals, shearwaters are intermediate, and oystercatchers exhibit the largest departure. Their empirical transport-effort frontiers therefore fall at different distances from a common reduced bound, allowing proximity to the minimum-cost soaring regime to be estimated directly from trajectory data.

## Concluding Remarks.

From Leonardo da Vinci’s account of birds drawing energy from wind shear to Rayleigh’s 1883 mechanical argument, and from there to more modern analyses of soaring energetics and optimal trajectories, dynamic soaring has been treated as a problem in which atmospheric structure enters the transport budget of flight (1, 2, 13, 14). The step taken here is to reduce that problem at the scale of transport, so that once trajectories are expressed in normalized speed and effort, birds with different flight modes can be placed in a common plane, and their empirical frontiers can be compared with the lower bound of the HJB model. Thus, the ordering of albatrosses, shearwaters, and oystercatchers acquires a mechanical interpretation because it indexes how much of the cost of transport can be offset by atmospheric input before sustained propulsion becomes dominant. The HJB bound is best read as a reduced benchmark rather than a fit-free law, since its coefficients are estimated from the albatross frontier, and the plotted effort ordinate is a VeDBA-based surrogate rather than direct mechanical work. Field observations already indicate limits beyond the reduced energetic minimum, a leveling-off of wandering albatross across-wind mean airspeed near 20 m s^−1^ at higher wind speeds, and a take-off cost that depends on both wind and waves (5, 9). Our reduced physical coordinates and theory-shaped frontiers offer a forward-looking route from tracking archives to mechanistic inference, and the same language should extend naturally to wind reconstruction from trajectories, animal-borne ocean sensing, and eventually to engineered dynamic-soaring vehicles.

## Supplementary Material

Supplement 1

## Data Availability

All study data are included in the article and/or at an online data repository – https://github.com/bhamla-lab/albatross-gonzalez-2026.
