# Supplementary material for: Seabird trajectories map onto a reduced optimal-control bound for dynamic soaring
Source: ArXiv. 2026 Apr 15:arXiv:2604.14310v1. Preprint. [Version 1] (PMC13105242)
Supplement: Supplement 1 [file NIHPP2604.14310v1-supplement-1.pdf]

Supplementary Information Appendix: Seabird trajectories reveal a physical limit to dynamic soaring

Louis González[1,2,3] and Saad Bhamla[1,2,3]

[1]School of Chemical & Biomolecular Engineering, Georgia Institute of Technology, Atlanta, USA
[2]BioFrontiers Institute, University of Colorado, Boulder, USA
[3]Department of Chemical & Biological Engineering, University of Colorado, Boulder, USA

Extended Methods

S1. Mechanical framework for dynamic soaring

We model the bird as an effective glider moving through an atmospheric flow. Let $\boldsymbol{x}(t)$ denote position in an Earth-fixed frame and let

$$\boldsymbol{u}(t) = \dot{\boldsymbol{x}}(t) \tag{1}$$

be the ground velocity. Let $\boldsymbol{w}(\boldsymbol{x}, t)$ denote the local wind velocity field. The air-relative velocity is then

$$\boldsymbol{v}(t) = \boldsymbol{u}(t) - \boldsymbol{w}(\boldsymbol{x}(t), t). \tag{2}$$

The specific mechanical energy in the ground frame is

$$E(t) = \frac{1}{2}|\boldsymbol{u}(t)|^2 + gz(t), \tag{3}$$

where $z(t)$ is altitude, and g is gravitational acceleration.

The translational equation of motion is

$$m\dot{\boldsymbol{u}} = \boldsymbol{F}_{\text{aero}} + \boldsymbol{F}_{\text{muscle}} - mg\,\hat{\boldsymbol{z}}, \tag{4}$$

where $\boldsymbol{F}_{\text{aero}}$ is the total aerodynamic force and $\boldsymbol{F}_{\text{muscle}}$ is the net propulsive force associated with active flapping or other muscular efforts. Taking the dot product with $\boldsymbol{u}$ gives

$$m\,\boldsymbol{u} \cdot \dot{\boldsymbol{u}} = \boldsymbol{u} \cdot \boldsymbol{F}_{\text{aero}} + \boldsymbol{u} \cdot \boldsymbol{F}_{\text{muscle}} - mg\,\dot{z}. \tag{5}$$

Using

$$\frac{d}{dt}\left(\frac{1}{2}|\boldsymbol{u}|^2\right) = \boldsymbol{u} \cdot \dot{\boldsymbol{u}}, \qquad \frac{d}{dt}(gz) = g\dot{z}, \tag{6}$$

we obtain

$$\frac{dE}{dt} = \frac{1}{m}\boldsymbol{u} \cdot \boldsymbol{F}_{\text{aero}} + \frac{1}{m}\boldsymbol{u} \cdot \boldsymbol{F}_{\text{muscle}}. \tag{7}$$

We now decompose the aerodynamic force into lift and drag,

$$\boldsymbol{F}_{\text{aero}} = \boldsymbol{L} + \boldsymbol{D}. \tag{8}$$

By definition,

$$\boldsymbol{L} \cdot \boldsymbol{v} = 0, \qquad \boldsymbol{D} = -D\,\hat{\boldsymbol{v}}, \qquad \hat{\boldsymbol{v}} = \frac{\boldsymbol{v}}{|\boldsymbol{v}|}, \qquad D \geq 0. \tag{9}$$

Since $\boldsymbol{u} = \boldsymbol{v} + \boldsymbol{w}$, we have

$$\boldsymbol{u} \cdot \boldsymbol{L} = (\boldsymbol{v} + \boldsymbol{w}) \cdot \boldsymbol{L} = \boldsymbol{w} \cdot \boldsymbol{L}, \tag{10}$$

and

$$\boldsymbol{u} \cdot \boldsymbol{D} = (\boldsymbol{v} + \boldsymbol{w}) \cdot (-D\hat{\boldsymbol{v}}) = -D|\boldsymbol{v}| + \boldsymbol{w} \cdot \boldsymbol{D}. \tag{11}$$

Therefore,

$$\boldsymbol{u} \cdot \boldsymbol{F}_{\text{aero}} = \boldsymbol{w} \cdot (\boldsymbol{L} + \boldsymbol{D}) - D|\boldsymbol{v}|. \tag{12}$$

Substituting into the energy balance yields

$$\frac{dE}{dt} = \frac{1}{m} \boldsymbol{w} \cdot (\boldsymbol{L} + \boldsymbol{D}) - \frac{D|\boldsymbol{v}|}{m} + \frac{1}{m} \boldsymbol{u} \cdot \boldsymbol{F}_{\text{muscle}}. \tag{13}$$

We therefore identify

$$\frac{dE}{dt} = P_{\text{wind}} - P_{\text{drag}} + P_{\text{muscle}}, \tag{14}$$

with

$$P_{\text{wind}} \equiv \frac{1}{m} \boldsymbol{w} \cdot (\boldsymbol{L} + \boldsymbol{D}), \qquad P_{\text{drag}} \equiv \frac{D|\boldsymbol{v}|}{m}, \qquad P_{\text{muscle}} \equiv \frac{1}{m} \boldsymbol{u} \cdot \boldsymbol{F}_{\text{muscle}}. \tag{15}$$

Dynamic soaring corresponds to the regime in which sustained transport is achieved primarily by positive environmental work P_{wind} rather than by large sustained muscular input.

S2. Integrated work balance and empirical wind-harvest estimate

Integrating the instantaneous energy balance over a time interval $[t_0, t_1]$ gives

$$E(t_1) - E(t_0) = \int_{t_0}^{t_1} P_{\text{wind}}(t)\,dt - \int_{t_0}^{t_1} P_{\text{drag}}(t)\,dt + \int_{t_0}^{t_1} P_{\text{muscle}}(t)\,dt. \tag{16}$$

Define

$$\Delta E \equiv E(t_1) - E(t_0), \tag{17}$$

$$W_{\text{wind}} \equiv \int_{t_0}^{t_1} P_{\text{wind}}(t)\,dt, \qquad W_{\text{drag}} \equiv \int_{t_0}^{t_1} P_{\text{drag}}(t)\,dt, \qquad W_{\text{muscle}} \equiv \int_{t_0}^{t_1} P_{\text{muscle}}(t)\,dt. \tag{18}$$

Then

$$\Delta E = W_{\text{wind}} - W_{\text{drag}} + W_{\text{muscle}}. \tag{19}$$

For windows dominated by gliding rather than sustained flapping, we approximate

$$W_{\text{muscle}} \approx 0, \tag{20}$$

which gives

$$W_{\text{wind}} \approx \Delta E + W_{\text{drag}}. \tag{21}$$

This is the cumulative energy-harvest relation used throughout the empirical ledger. In the code, the signed drag contribution is stored as a negative cumulative quantity,

$$W_{\text{drag}}(t) = \int_{t_0}^{t} \left(-P_{\text{drag}}(s)\right)\,ds, \tag{22}$$

so that the inferred harvest is implemented as

$$W_{\text{harvest}}(t) = (E(t) - E(t_0)) - W_{\text{drag}}(t). \tag{23}$$

Because $W_{\text{drag}}(t) \leq 0$, this is algebraically equivalent to adding the magnitude of the dissipative loss.

S3. Drag model and species-specific parameters

To estimate aerodynamic dissipation from trajectory data, we use a quasi-steady gliding approximation. By definition,

$$\frac{L}{D} = \frac{L}{D}, \tag{24}$$

so

$$D = \frac{L}{L/D}. \tag{25}$$

For sustained gliding outside strong transients,

$$L \approx mg, \tag{26}$$

hence

$$D \approx \frac{mg}{L/D}. \tag{27}$$

The specific drag power is then

$$P_{\text{drag}} = \frac{D|\boldsymbol{v}|}{m} \approx \frac{g|\boldsymbol{v}|}{L/D}. \tag{28}$$

Because the trajectories are analyzed in the ground frame and the surrounding wind field is not reconstructed pointwise, we replace airspeed by the measured speed used in the ledger:

$$\widehat{P}_{\text{drag}} \approx \frac{g\,u}{L/D}, \qquad u = |\boldsymbol{u}|. \tag{29}$$

In the implementation, this appears with a negative sign as

$$P_{\text{drag}}^{\text{signed}} = -\frac{g\,u}{L/D}. \tag{30}$$

The species-specific values used in the figure-generation code were

$$(L/D)_{\text{alb}} = 22, \qquad (L/D)_{\text{shw}} = 18, \qquad (L/D)_{\text{oyst}} = 10. \tag{31}$$

These values are not random. Wandering albatrosses can glide at a maximum straight-flight ratio of about 21.2, so we chose a code-level value of $L/D = 22$ as a rounded figure that fits existing literature [1]. The oystercatcher value is set lower as a cautious, general comparison. Wind-tunnel tests on birds with similar Reynolds numbers show maximum lift-to-drag ratios between 10 and 12.6 for trained birds [2, 3]. In contrast, non-gliding specialists show much lower ratios. By selecting $L/D = 10$, we place the oystercatcher in a reasonably low-efficiency category rather than giving it soaring abilities similar to an albatross. The shearwater value is considered to be between. This reflects the ecological role of shearwaters as flap-gliding seabirds. They can utilize shear-powered flight but are not as specially built for extreme gliding as wandering albatrosses. For the shearwater comparison, we used an effective value of $L/D = 18$. This choice is an estimate since there are no direct aerodynamic measurements for Cory's shearwaters. They are dynamic soarers with a higher L/D than oystercatchers, but they are not as large as wandering albatrosses.

S4. Interpretation of ground speed versus airspeed

The recorded kinematic quantities are fundamentally ground-referenced. For the wandering albatross dataset, the code uses Doppler eastward and northward components,

$$u(t) = \sqrt{u_E^2(t) + u_N^2(t)}, \qquad u_E(t) = \texttt{s2east}(t), \qquad u_N(t) = \texttt{s2north}(t), \tag{32}$$

and altitude

$$z(t) = \texttt{alt}(t). \tag{33}$$

Thus, the empirical energy ledger is a ground-frame ledger,

$$E(t) = \frac{1}{2}u^2(t) + gz(t). \tag{34}$$

This does not eliminate the role of wind. Instead, it is precisely in the ground frame that a moving atmosphere can do nonzero work on the bird. What is not available in the present dataset-level analysis is a full reconstruction of the ambient wind field along each trajectory. For that reason, the quantity we infer from the ledger is best interpreted as an effective cumulative atmospheric input,

$$\widehat{W}_{\text{wind}} = \Delta E + \widehat{W}_{\text{drag}}, \tag{35}$$

rather than a direct pointwise measurement of the aerodynamic work of the wind field.

S5. Datasets

Wandering albatrosses. The primary dataset is the Dryad archive "Behavioral datasets of wandering albatrosses collected at Possession Island, Crozet, France, in 2019 and 2020." The repository includes raw GPS (`G`), acceleration (`A`), pressure-temperature (`P`), and geomagnetism (`M`) files. The README states that the Ninja-scan recorders sampled triaxial acceleration and angular velocity at 100 Hz, GPS position and Doppler velocity at 5 Hz, and temperature, pressure, and geomagnetism at 6 Hz. Data were collected at Possession Island in the Crozet Archipelago, South Indian Ocean, during 2019 and 2020. In summary, 21 birds were tagged in 2019 and 24 in 2020, with one isolated deployment in 2020 failing to record correctly, yielding 44 valid individuals overall. The analysis code pairs `*_A.csv` and `*_G.csv` files by bird identifier and processes all valid matched pairs.

Cory's shearwaters. The comparison shearwater dataset came from the Movebank data package provided by the authors: https://doi.org/10.5441/001/1.nf80477p. The linked accession corresponds to the displaced-bird Cory's shearwater study from the Azores, which included a total of 24 birds. Twelve birds were released in 2010, and 12 were released in 2011. The data come from two flat files, `coryshearwater-gpsonly.csv` and `coryshearwater-accelerationonly.csv`. The filenames do not include the year label. Therefore, our pipeline does not allow for the exact year subset to be retrieved easily. The shearwater comparator was taken from a single group in the linked Movebank accession.

Using a single annual shearwater cohort is justifiable in this study. The shearwaters do not fit the dynamic soaring limit. They are used only to establish an intermediate flap-gliding flight economy in the reduced phase space. Combining multiple years would mainly expand the comparator cloud by adding more interannual environmental differences while also giving more weight to that comparison class compared to the albatross reference. Since the reduced variables are normalized within the dataset by V_{base} and $\mathcal{E}_0$, the role of the shearwater class as an intermediate comparator does not rely on pinpointing year-to-year changes in detail.

Eurasian oystercatchers. The oystercatcher comparison dataset is the Zenodo record "O_VLIELAND - Eurasian oystercatchers (*Haematopus ostralegus*) breeding and wintering on Vlieland (the Netherlands)," DOI https://doi.org/10.5281/zenodo.10053988. According to the Zenodo metadata, the study was operational from 2016 to 2021 and includes 103 tagged individuals. The record contains year-wise GPS and acceleration files exported from Movebank as a frictionless data package. The oystercatcher comparison explicitly uses `O_VLIELAND-gps-2016.csv` and `O_VLIELAND-acceleration-2016.csv`. The reference-data table associated with that package contains 20 deployments in 2016, corresponding to 20 unique animal identifiers, so the single-year oystercatcher comparator used is a 20-bird subset.

S6. Preprocessing for the albatross population-level analysis

For the global wandering albatross analysis, our pipeline reads paired acceleration and GPS files, parses timestamps into seconds, and aligns the two streams on the GPS time base. The GPS stream is filtered to

remove entries with zero latitude or longitude. The median positive GPS time increment defines the effective GPS sampling interval

$$\Delta t_{\mathrm{GPS}} = \mathrm{median}\left\{t_{k+1} - t_k : t_{k+1} > t_k\right\}. \tag{36}$$

Acceleration components are then linearly interpolated onto the GPS times.

Dynamic body acceleration is computed by subtracting a low-frequency gravitational component from each interpolated acceleration channel. Specifically, each channel is low-pass filtered with a fourth-order Butterworth filter at the cutoff frequency

$$f_c = 0.2 \text{ Hz}, \tag{37}$$

and the dynamic component is defined as the residual after subtraction. The resulting vectorial dynamic body acceleration is

$$\mathrm{VeDBA}(t) = \sqrt{\left(a_x - a_x^{\mathrm{lp}}\right)^2 + \left(a_y - a_y^{\mathrm{lp}}\right)^2 + \left(a_z - a_z^{\mathrm{lp}}\right)^2}. \tag{38}$$

Ground speed is reconstructed from the Doppler eastward and northward components,

$$u(t) = \sqrt{u_E^2(t) + u_N^2(t)}, \tag{39}$$

and heading is

$$\psi(t) = \mathrm{atan2}(u_N(t), u_E(t)). \tag{40}$$

The turn rate is then computed from the unwrapped heading by finite differentiation,

$$\dot{\psi}(t) \approx \frac{d}{dt}\,\mathrm{unwrap}\,\psi(t). \tag{41}$$

S7. Windowing and window-level observables

Continuous albatross trajectories are partitioned into overlapping windows of duration

$$T_w = 120 \text{ s} \tag{42}$$

with step size

$$\Delta T = 30 \text{ s}. \tag{43}$$

A window is retained only if at least 90% of the speed values in that window are finite and if the mean ground speed satisfies

$$\overline{u} \geq 6.0 \text{ m/s}. \tag{44}$$

For energy-ledger quantities, the code additionally requires that at least 80% of altitude values within the window be finite.

For each retained window, the code computes a transport-progress statistic

$$\mathrm{progress} = \frac{1}{T_w}\sqrt{\left(\sum_{k\in w} u_E(t_k)\,\Delta t\right)^2 + \left(\sum_{k\in w} u_N(t_k)\,\Delta t\right)^2}, \tag{45}$$

the mean dynamic body acceleration

$$\overline{\mathrm{VeDBA}} = \frac{1}{N_w}\sum_{k\in w} \mathrm{VeDBA}(t_k), \tag{46}$$

and a dynamic-soaring spectral intensity defined as the Welch bandpower of the turn-rate signal in the frequency band

$$f \in [0.03,\, 0.15] \text{ Hz}. \tag{47}$$

That is,

$$P_{\mathrm{DS}} = \int_{0.03}^{0.15} S_{\dot{\psi}}(f)\, df, \tag{48}$$

where $S_{\dot{\psi}}(f)$ is the Welch estimate of the turn-rate power spectral density.

Within each window, the energy ledger uses a Savitzky–Golay filter with polynomial order

$$p = 2 \tag{49}$$

and window length

$$T_{\mathrm{SG}} = 3.0 \text{ s} \tag{50}$$

applied separately to speed and altitude before constructing

$$E(t) = \frac{1}{2} u_{\mathrm{sm}}^2(t) + g z_{\mathrm{sm}}(t). \tag{51}$$

The code stores three window-level energy quantities:

$$\overline{P}_{\mathrm{harvest}} = \frac{W_{\mathrm{harvest}}(t_1) - W_{\mathrm{harvest}}(t_0)}{T_w}, \tag{52}$$

$$\overline{P}_{\mathrm{drag}} = \frac{W_{\mathrm{drag}}(t_1) - W_{\mathrm{drag}}(t_0)}{T_w}, \tag{53}$$

and

$$\Delta E_{\mathrm{rel}} = E_{\mathrm{rel}}(t_1) - E_{\mathrm{rel}}(t_0). \tag{54}$$

S8. Empirical Pareto construction for the effort–progress plane

The population-level Pareto plane used for the albatross analysis (Figure 1d) takes

$$X_{\mathrm{emp}} = \mathrm{progress}_{\mathrm{m/s}}, \qquad Y_{\mathrm{emp}} = \overline{\mathrm{VeDBA}}. \tag{55}$$

After removing windows with non-finite values, the frontier is estimated by binning the X_{emp} axis into

$$N_{\mathrm{bins}} = 30 \tag{56}$$

bins between the 2nd and 98th percentiles of the observed progress values. In each populated bin, the lower-envelope frontier ordinate is taken to be the

$$q = 0.10 \tag{57}$$

quantile of the VeDBA values. The resulting binned frontier points are then fit by the empirical envelope

$$Y_{\mathrm{env}}(x) = a + \frac{b}{x} + cx^2 \tag{58}$$

using linear least squares in the basis $\{1,\, 1/x,\, x^2\}$. This fit is used only as a smooth visual representation of the observed lower envelope.

S9. Comparative reduced phase space and derivation of the HJB limit

A second representation is used to compare albatrosses, Cory's shearwaters, and oystercatchers in a common reduced phase space (Figure 2a). For the comparison species, GPS and acceleration streams are merged by the nearest timestamp after independent parsing. For shearwaters, raw burst acceleration strings are unpacked into triaxial acceleration samples using the nominal burst frequency

$$f_{\mathrm{acc}}^{\mathrm{shw}} = 18.74 \text{ Hz}, \tag{59}$$

with a centered rolling mean of window size 19 samples used to estimate the static component. GPS and acceleration are merged with a tolerance of 60 s. For oystercatchers, we use acceleration channels `tilt-x`, `tilt-y`, and `tilt-z`, a centered rolling mean of size 10 samples, and a merge tolerance of 2 s.

After the merge, rows are retained only if

$$1.0 < \texttt{ground-speed} < 30.0 \text{ m/s}. \tag{60}$$

For a given species dataset, let V_{obs} denote the retained ground-speed values and let $\mathcal{E}_{\text{obs}}$ denote the corresponding VeDBA values. The reduced variables are then defined by

$$\mathcal{E}_0 = \text{percentile}_5(\mathcal{E}_{\text{obs}}), \tag{61}$$

$$V_{\text{base}} = \text{median}\,\{V_{\text{obs}} : \mathcal{E}_{\text{obs}} < \text{percentile}_{15}(\mathcal{E}_{\text{obs}})\}\,,, \tag{62}$$

$$X = \frac{V_{\text{obs}}}{V_{\text{base}}}, \qquad Y = \frac{\mathcal{E}_{\text{obs}} - \mathcal{E}_0}{\mathcal{E}_0}. \tag{63}$$

Thus, X is a reduced transport speed and Y is a normalized excess effort above a low-effort baseline.

For the albatross population, the same transformation is applied bird-by-bird to the window-level quantities `progress_m_s` and `VeDBA_mean`. We first restrict to values satisfying

$$1 < \text{progress} < 35, \qquad 0.1 < \overline{\text{VeDBA}} < 10, \tag{64}$$

and then sets

$$\mathcal{E}_0 = \text{percentile}_5(\overline{\text{VeDBA}}), \tag{65}$$

$$V_{\text{base}} = \text{clip}\big(\text{median}\,\big\{\text{progress} : \overline{\text{VeDBA}} < \text{percentile}_{15}(\overline{\text{VeDBA}})\big\}\,,\, 10,\, 20\big)\,. \tag{66}$$

Species-specific frontiers in the reduced plane are then extracted as the 10th percentile lower envelope in bins of X, using 60 steps for albatrosses and 40 steps for the two comparison classes.

We now derive the theoretical HJB limit curve in Figure 2a. Consider one-dimensional progress along a prescribed migration or return direction. Let $s(t)$ denote the remaining along-track distance to be covered. We take

$$\dot{s}(t) = -V(t), \qquad V(t) > 0, \tag{67}$$

so the control variable is the transport speed $V(t)$. The quantity to be minimized is the total specific muscular work needed to traverse the remaining distance. Define the value function

$$J(s) = \inf_{V(\cdot)} \int_0^T p_{\text{muscle}}\big(V(t)\big)\, dt, \tag{68}$$

subject to

$$\dot{s}(t) = -V(t), \qquad s(0) = s, \qquad s(T) = 0. \tag{69}$$

Here $p_{\text{muscle}}(V)$ is the specific muscular power required to sustain transport speed V after accounting for aerodynamic losses and for energy harvested from the wind.

To obtain an analytically tractable reduced model, we decompose p_{muscle} into three parts.

First, induced drag decreases with increasing speed. Writing $D_i \propto 1/V^2$ in quasi-steady flight gives an induced-power contribution

$$p_i(V) = \frac{A}{V}, \tag{70}$$

where $A > 0$ is an effective constant incorporating body weight and wing geometry.

Second, parasite drag increases quadratically with speed, $D_p \propto V^2$, so the corresponding power scales as

$$p_p(V) = BV^3, \tag{71}$$

with $B > 0$, an effective parasite-drag coefficient.

Third, the dynamic-soaring harvest term is taken to scale as

$$p_w(V) = \Gamma V^2, \tag{72}$$

with $\Gamma > 0$. This scaling follows from a simple cycle argument. If one dynamic-soaring cycle extracts an amount of energy proportional to the shear encountered over the cycle, then the energy gained per cycle scales as $\Delta E_{\mathrm{cyc}} \sim mV\Delta U$, where ΔU is an effective shear increment. If the spatial extent of a cycle is ℓ, then the cycle frequency scales as V/ℓ. The mean harvested power is therefore

$$P_{\mathrm{harvest}} \sim \Delta E_{\mathrm{cyc}} \times \frac{V}{\ell} \propto V^2. \tag{73}$$

The coefficient Γ absorbs the effective wind shear, cycle geometry, and conversion efficiency.

The net specific muscular power is therefore modeled as

$$p_{\mathrm{muscle}}(V) = \frac{A}{V} + BV^3 - \Gamma V^2. \tag{74}$$

Because the problem is stationary in the remaining distance variable s, the Hamilton–Jacobi–Bellman equation is

$$0 = \inf_{V>0} \{p_{\mathrm{muscle}}(V) + J_s(s)\,\dot{s}\} = \inf_{V>0} \{p_{\mathrm{muscle}}(V) - VJ_s(s)\}, \tag{75}$$

with boundary condition

$$J(0) = 0. \tag{76}$$

In a homogeneous environment, the optimal cost is extensive in distance, so we set

$$J(s) = \lambda s, \tag{77}$$

where λ is the minimum specific energetic cost per unit distance. Then

$$J_s(s) = \lambda, \tag{78}$$

and the HJB equation becomes

$$0 = \inf_{V>0} \left\{ \frac{A}{V} + BV^3 - \Gamma V^2 - \lambda V \right\}. \tag{79}$$

Dividing by $V > 0$ gives

$$\lambda = \inf_{V>0} \left\{ \frac{A}{V^2} + BV^2 - \Gamma V \right\}. \tag{80}$$

This expression is the reduced optimal-control bound. It states that the minimum cost per unit distance is obtained by minimizing the competition among an induced-drag term A/V^2, a parasite-drag term BV^2, and a wind-harvest term $-\Gamma V$.

The stationary point satisfies

$$\frac{\mathrm{d}}{\mathrm{d}V} \left(\frac{A}{V^2} + BV^2 - \Gamma V \right) = 0, \tag{81}$$

that is,

$$-\frac{2A}{V^3} + 2BV - \Gamma = 0. \tag{82}$$

Multiplying by V^3 yields

$$2BV^4 - \Gamma V^3 - 2A = 0, \tag{83}$$

which determines the HJB-optimal transport speed in the reduced model.

To map this result onto the plotted reduced coordinates, we write

$$V = V_{\mathrm{base}} X \tag{84}$$

and introduce a characteristic cost scale C_0. Then

$$\frac{1}{C_0}\left(\frac{A}{V^2} + BV^2 - \Gamma V\right) = \frac{A}{C_0 V_{\mathrm{base}}^2}\frac{1}{X^2} + \frac{BV_{\mathrm{base}}^2}{C_0}X^2 - \frac{\Gamma V_{\mathrm{base}}}{C_0}X. \tag{85}$$

Defining

$$a \equiv \frac{A}{C_0 V_{\mathrm{base}}^2}, \qquad b \equiv \frac{BV_{\mathrm{base}}^2}{C_0}, \qquad W \equiv \frac{\Gamma V_{\mathrm{base}}}{C_0}, \tag{86}$$

we obtain the reduced HJB form

$$Y_{\mathrm{HJB}}(X) = \frac{a}{X^2} + bX^2 - WX. \tag{87}$$

The displayed curve is clipped at zero,

$$Y_{\mathrm{HJB}}(X) = \max\Big(0, \frac{a}{X^2} + bX^2 - WX\Big), \tag{88}$$

which reflects the fact that the plotted ordinate is a normalized excess-effort quantity relative to a low-effort baseline. Negative reduced costs are therefore not displayed as negative effort. In practice, the parameters (a, b, W) are estimated by nonlinear least squares from the albatross frontier. Accordingly, our reduced HJB-limit is justified by the form of the reduced optimal-control derivation above, while the numerical parameter values are determined empirically from the data.

S10. Energy-ledger comparison figure

We also construct a comparative cumulative-energy figure for all three species. For each candidate segment, a speed column is chosen from the available fields in the order

$$\texttt{ground-speed}, \quad \texttt{speed}, \quad \texttt{v}, \quad \texttt{s2north} \tag{89}$$

with

$$u = \sqrt{\texttt{s2north}^2 + \texttt{s2east}^2} \tag{90}$$

used when both Doppler components are present. Altitude is chosen from the first available one

$$\texttt{height-above-ellipsoid}, \quad \texttt{alt}, \quad \texttt{altitude}. \tag{91}$$

Rows with implausible values are removed using the hard thresholds

$$0 \le u < 50 \text{ m/s}, \qquad -50 < z < 200 \text{ m}. \tag{92}$$

If fewer than 60 seconds of data remain at the nominal sampling rate, the segment is discarded. Otherwise, only the first 120 seconds are retained. Speed and altitude are then smoothed with a Savitzky-Golay filter of order 2 and a window length of approximately 5 seconds before computing

$$E(t) = \frac{1}{2}u_{\mathrm{sm}}^2(t) + g z_{\mathrm{sm}}(t), \tag{93}$$

$$W_{\mathrm{drag}}(t) = \sum_{k \le t}\left(-\frac{g\, u_{\mathrm{sm}}(t_k)}{L/D}\right)\Delta t, \tag{94}$$

$$W_{\mathrm{harvest}}(t) = (E(t) - E(0)) - W_{\mathrm{drag}}(t). \tag{95}$$

The mean harvested power reported for each segment is

$$\overline{P}_{\mathrm{harvest}} = \frac{W_{\mathrm{harvest}}(t_{\mathrm{end}})}{t_{\mathrm{end}}}. \tag{96}$$

S11. Limitations and scope of interpretation

Some key things to note when looking at our results and modeling efforts are the following:

First, our empirical ledger cannot differentiate between ground speed and airspeed. As a result, the values W_{drag} and W_{harvest} may be thought of as effective energetic measures rather than faithful replicas of aerodynamic work.

Second, our energy ledger makes the assumption that gliding drag being constant, which means that variations in posture, bank angle, Reynolds number, or transient loading are not considered. We clarify the explicit reasoning behind our energy ledger.

Third, the reduced HJB function is derived based on a stationary optimal control problem where we minimize the costs per unit distance. In reality, its parameters depend on the albatross empirical frontier, and the vertical axis denotes a normalized estimate of the effort proxy VeDBA rather than the actual mechanical work done. We would call it the reduced HJB limit within the dimensionless effort vs. speed plane rather than an entire first principles aerodynamic closure of the flight dynamics.

Finally, the raw-sensor pipeline applied to analyzing our albatross data is distinct from what we use in the case of comparison datasets. Although the latter are treated to yield the comparative reduced variables, there will still be differences in how raw sensor values are processed.

References

[1] Colin J Pennycuick. *Modelling the flying bird.* Vol. 5. Elsevier, 2008.

[2] Vance A Tucker and Carlton Heine. "Aerodynamics of gliding flight in a Harris' hawk, Parabuteo unicinctus". In: *Journal of Experimental Biology* 149.1 (1990), pp. 469–489.

[3] Mikael Rosén and Anders Hedenström. "Gliding flight in a jackdaw: a wind tunnel study". In: *Journal of Experimental Biology* 204.6 (2001), pp. 1153–1166.
